# Effect of Centrifugal Shot Peening on the Surface Properties of Laser-Cut C45 Steel Parts

**DOI:** 10.3390/ma12213635

**Published:** 2019-11-05

**Authors:** Agnieszka Skoczylas, Kazimierz Zaleski

**Affiliations:** Department of Production Engineering, Faculty of Mechanical Engineering, Lublin University of Technology, 36 Nadbystrzycka Street, 20-618 Lublin, Poland; k.zaleski@pollub.pl

**Keywords:** laser cutting, centrifugal shot peening, surface roughness, microstructure, microhardness, residual stress

## Abstract

This article presents the results of experimental studies of the impact of centrifugal shot peening parameters on the roughness, microstructure, and microhardness of the surface layer of laser-cut C45 steel parts. Residual stress distributions and the presence of iron oxides on the surface of these elements were also examined. Centrifugal shot peening tests were performed on an FV-580a vertical machining center while using a specially designed peening head. The parameters that were varied during centrifugal shot peening included tangential speed of the tool v_g_ and feed rate v_f_. The use of centrifugal shot peening for finish machining of laser-cut C45 steel parts allowed for obtaining a four-fold reduction in the surface roughness parameters Ra and Rz. As a result of shot peening, the geometrical structure of the surface of the steel parts was modified and it acquired new beneficial features, such as large values of the rounding radii of the micropeaks and high material ratios (Rmr_max_ = 92%). At the same time, the surface layer was hardened (microhardness increased by 16%) and a compressive residual stress layer was produced on the surface of the workpieces. Additionally, as the shot impacted the processed surface, combustion products were “blasted” or “sheared” off it. Shot peening using the proposed technique can be successfully performed while using CNC machines.

## 1. Introduction

Laser cutting is a process in which a (continuous or pulsed) laser beam melts, simultaneously melts and evaporates, or melts and/or burns away the material in the cut (the kerf). It is a thermal cutting process that uses a large amount of energy that is focused on a small area [1]. Along with the laser beam, a stream of gas is emitted coaxially; the gas might be reactive to the material being cut (oxygen, air) or it might be an inert gas (nitrogen, argon). The presence of a reactive gas in the kerf produces an exothermic reaction, which effectively increases the power of the laser, allowing it to cut material of considerable thickness [2,3]. An inert gas is used to remove molten material and it also acts as a cooling and protective agent for the optical system of the laser cutter [3,4].

Laser technologies can be used to section most engineering materials, e.g. unalloyed steel [1,5], stainless steel [4,6], aluminum alloys [7], nickel alloys [8], carbon fiber reinforced polymer composites [9], and also natural materials. The laser cutting of metallic materials is widely used in the automotive, chemical, marine, and aviation industries [10]. The strengths of this material separation technology include a high cutting speed (many times faster than the speeds used during machining), satisfactory dimensional accuracy, and low roughness of the cut surface, as well as a high level of automation and flexibility [1,11,12].

During laser cutting, the coherent laser beam might generate imperfections on the cut faces and edges. Imperfections, according to the EN ISO 17658: 2015 standard, are irregularities or deviations from the specified shape or location of cut. The laser cutting process directly causes them (no adverse phenomena resulting from external stresses or deformations are taken into account). 

The quality of the surface and the course of the cutting process depend on many factors with different levels of control. The most important of them include laser power, laser type and operation mode, position of laser beam focus relative to the surface of the workpiece, cutting speed, and assist gas type and pressure [10].

The quality of laser-cut edges and surfaces is evaluated quantitatively in accordance with EN ISO 9013:2017. In industrial practice, surface roughness, width of the heat affected zone, material phase changes, residual stresses in the vicinity of the kerf, and the formation of dross on the lower edges of the kerf define the quality of laser-cut surfaces [1,9,11,13].

Surface roughness in laser cutting is the result of the overlapping effects of the thermal and hydrodynamic processes that are characteristic of laser processing and vibrations of the workpiece caused by the high-pressure gas jet [3]. The surface of the workpiece can be divided into two areas of different shape and quality. These zones are separated from each other by the so-called boundary layer separation (BLS). One zone is the laser beam entrance area and the other is the laser beam exit area; the latter has a much higher surface roughness [11,14]. A characteristic feature of the laser cutting process is the formation of striations on the cut surface. The striations pattern forms as a consequence of hydrodynamic flow of molten material, laser power fluctuation, gas flow fluctuation, and laser head oscillation [15,16].

During laser cutting, the heat that is delivered to the material and the high temperature gradient create a thin film of material with variable properties, called the heat affected zone, which is characterized by a small thickness, but locally, especially at the edges and corners, has high hardness. The differences in hardness may be associated with a non-uniform distribution of energy at beam cross-section and differences in heat dissipation rates [17,18].

The high temperature in the cutting zone gives rise to thermal stresses. Heating of the material followed by rapid cooling with the assist gas leads to material shrinkage and phase changes. The dynamics of phase changes at different cooling rates lead to the formation of tensile residual stresses. The value and distribution of thermal stresses depend on the thermal conductivity of the material being cut [11,13].

Non-alloy steel characteristically forms a thin surface layer of iron oxide phases FeO and Fe_3_O_4_, which change the color of the surface to bluish [19,20,21]. The resultant particles are about 50 µm in size and they most often have the form of iron spheres that are coated with iron oxide [3].

The presence of various imperfections on the surfaces and edges of laser-cut parts and the presence of drops of solidified material at their bottom edges calls for finishing treatment. Scale and outflow can be removed with hand files, wire brushes, deburring machines, and belt sanders. Mechanical grinding [22] and smoothing with diamond tools [23] can improve the geometrical structure of a laser-cut surface. 

Mechanical grinding allows for reducing surface roughness and the thickness of the hardened layer and introduces into the workpiece a state of tensile residual stress, which might lead to cracking of the surface. The resultant defects are loci of stress concentration and reduce the bearing capacity of the processed surface [22].

Smoothing with diamond tools allows for reducing surface roughness by several times. At the same time, it causes structural and phase changes in the material. As a result of smoothing, residual austenite is transformed into martensite. The surface layer is mechanically hardened. The use of a diamond tool is associated with the occurrence of sliding friction between the tool and the surface of the workpiece, which leads to an increase in temperature in the machining zone. Hardness in the edge zone is higher than that after laser cutting; discontinuities and microcracks also appear [23].

The condition of the surface and edges of a laser-cut semi-finished product does not allow or makes its use difficult in subsequent production stages. It is also difficult to apply paint coatings to it because of flaking. 

Centrifugal shot peening changes the properties of the surface layer of workpieces pre-machined by turning, milling, or grinding. Centrifugal shot peening of a pre-milled surface leads to the formation of a dimpled surface with elongated depressions that can retain additional lubricant [24]. The use of optimal centrifugal shot peening conditions during finish machining of turned C45 and 40H steel shafts, allows for obtaining a several-fold reduction in Ra. The positive effects of using shot peening for post-processing of machined parts encourage the use of centrifugal shot peening to modify the surface layer of laser-cut parts. 

Shot peening as a process used to finish machine parts allows for obtaining a surface that is characterized by a low roughness [25] and a large bearing area (material ratio). This is due to the more “streamlined” shape of the micro asperities that form during peening [25,26]. Indentations imparted to the shot-peened surface serve as “reservoirs” for lubricants, allowing the formation of a protective layer that increases the resistance of these surfaces to abrasive wear [27]. The surface texture formed during processing affects the energy state and the adhesive properties of the surface [28,29]. One positive effect of shot peening, apart from the fact that it improves the properties of the surface layer, is the rounding of the workpiece edges [30]. Centrifugal shot peening can also be combined in a single operation with grinding while using hybrid tools that enable the processing of difficult-to-cut materials, such as nickel and titanium alloys [31].

During shot peening, the concentration of crystal structure defects changes, as confirmed in tests that were carried out by annihilation techniques [32], as a result of which compressive stresses form in the surface layer of the workpiece [33]. Changes in the state of residual stress and the increase in the microhardness of the surface layer enhance the resistance of the material to abrasive and fatigue wear [34,35].

A review of the literature shows that the laser cut parts require finishing steps. The beneficial effect of shot peening on the condition of the surface of machined and ground parts is well-known. The aim of the present study was to assess the impact of centrifugal shot peening on the surface layer of C45 steel parts that were cut with laser.

## 2. Materials and Methods 

The tests were performed while using specimens of non-alloy C45 steel (in accordance with EN ISO 683-2:2018). This steel grade is used in the machine industry for the manufacture of medium-load machine and equipment parts, such as spindles, shafts, axles, and unhardened gears. Table 1 shows the chemical composition and strength properties of the steel tested (as per the technical specification sheet).

Rectangular specimens, 5 mm × 8 mm × 100 mm in size, were cut in a LASER Amada 3000 W laser cutter from Amada America Inc. (Düsseldorf, Germany), while using oxygen as the assist gas and standard parameters: cutting speed v = 1150 mm/min., power 2.15 kW, frequency f_Hz_ = 1280 Hz, assist gas pressure 0.06 MPa, and laser beam focus position +13 mm. 

Laser-cut specimens were finish-machined using centrifugal shot peening, which consisted in impacting the surface of a workpiece with shot propelled from openings radially arranged in a rotating peening head. After the head is set into rotational motion, the shot particles hit the surface of the workpiece under the influence of centrifugal force. Upon brief contact with the shot, the surface of the workpiece undergoes plastic deformation. Peening reduces the surface roughness and hardens the surface layer of the processed material. After centrifugal shot peening, the resulting machining marks on the surface are organised in the longitudinal and transverse direction, which allows for obtaining a surface with less roughness. It can be used to process axially symmetrical parts, flat surfaces, and surfaces with complex shapes. Moreover centrifugal shot peening allows for the treatment of elements with low rigidity.

**Figure 1:**
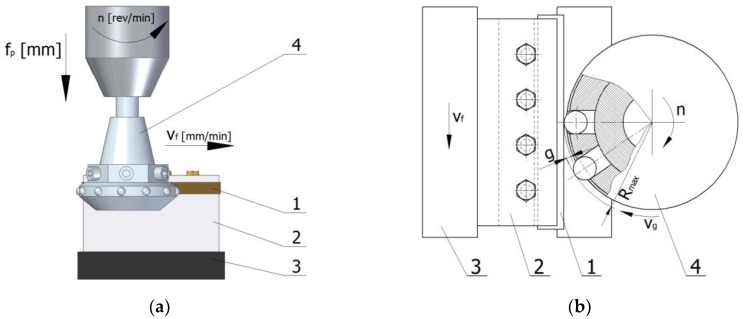
Scheme of centrifugal shot peening process on a vertical machining center (1—sample, 2—holder, 3—vice, 4—shot peening head): (**a**) shot peening kinematics; (**b**) principle of centrifugal shot peening (g—infeed, Rmax—maximum head radius).

Centrifugal shot peening tests were performed on an FV-580a (MOC Mechanicy Pruszków, Pruszków, Poland) vertical machining center while using a special peening head with an outer diameter of 70 mm with a blasting unit containing twelve (z_k_ =12) symmetrically arranged shot balls with a diameter d_k_ = 6.3 mm (Figure 1). During shot peening, specimen (1) was clamped in a holder (2) so that the peened surface was parallel to the spindle axis. The holder was secured in the jaws of a vice (3) that was mounted on the table of the machine tool, which moved at speed v_f_ during peening. The body of special head has radial holes, in which the balls move. In the guiding part the balls can move freely along the parting axis. On the body of the head a ring with radially arranged holes in which the balls are located is mounted. On the circumference of the ring the diameter of the holes is smaller than the diameter of the balls, which prevents the balls from falling out. The peening head (4) rotated with speed *n* and simultaneously moved in the cross-feed direction f_p_. When the head was set in rotational motion, shot particles were blasted from it under the influence of centrifugal force and they impacted the surface of the workpiece. Upon impact, the particles bounced off the peened surface and were retracted into the seats in the head at distance *g*, referred to as “head infeed to the workpiece”.

Centrifugal shot peening was performed while using Mobile Cut cooling lubricant (Mobile, Germany) at a constant infeed g = 0.5 mm and a constant cross-feed f_p_ = 0.08 mm; the number of passes was *i* = 1. The parameters that were varied in the experiments included

- tangential speed of shot peening head: v_g_ = 528–1143 m/min.

- feed rate: v_f_ = 1368–10488 mm/min.

**Figure 2:**
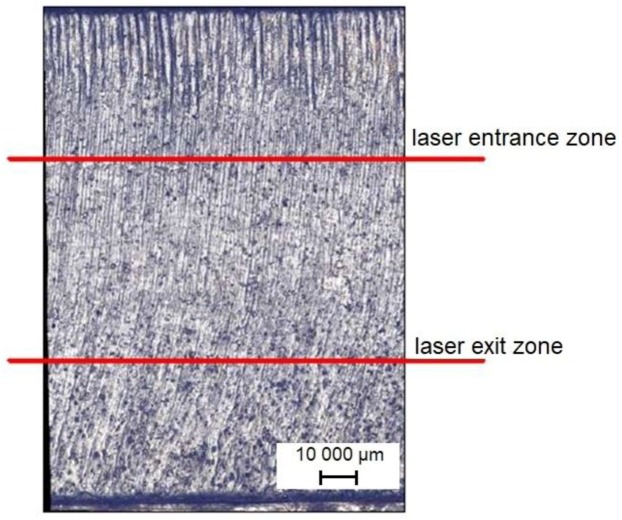
Surface view after laser cutting with marked roughness measurements point.

The measurements of surface roughness and three-dimensional (3D) topography were performed while using a Hommel-Etamic T8000RC 120–140 device (Jenoptik, Villingen-Schwenningen, Germany). According to EN ISO 9013:2017, measurements of surface roughness should be made at a distance equal to 1/3 of the thickness of the workpiece, from the upper cut edge, when the thickness of the workpiece is above 2 mm. Following the guidelines of the EN ISO 9013:2017 standard, roughness was measured at a distance of 1/3 of workpiece thickness from the upper cutting edge, in the so-called “laser entrance zone” and at a distance of 1/3 of workpiece thickness from the bottom cut edge, in the so-called “laser exit zone”. We decided to determine the roughness parameters in this lower area because the laser-cut parts had two characteristic zones with different roughness. Figure 2 schematically shows the place of roughness measurements. The same measurement scheme was used to measure the roughness of shot-peened specimens. Following the guidelines for roughness measurements outlined in EN ISO 4288:1997, the sampling length was set at lr = 2.5 mm. The material ratio Rmr was c = 2 µm below the highest peak of the roughness profile.

**Figure 3:**
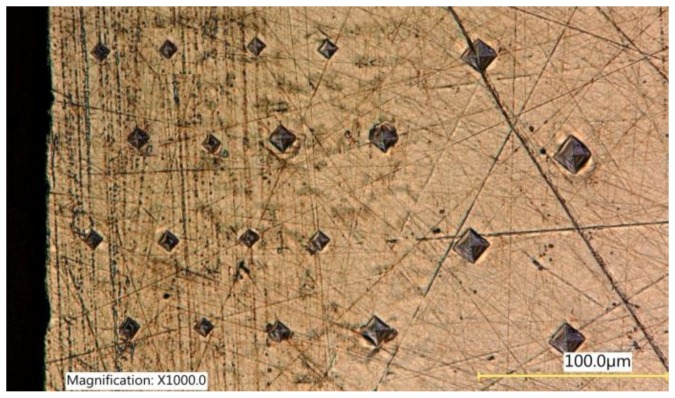
Schema of microhardness measurements.

The structure of the material was assessed while using a Nikon Eclipse MA 100 metallographic microscope (Nikon, London, UK). Microhardness measurements were made by the Vickers method in accordance with EN ISO 6507-1:2018 using transverse metallographic specimen surfaces that were prepared in a standard way. A LM Leco 700 at microhardness tester (Leco, San Jose, MI, USA) was used at an indenter load of 50 g (0.05 HV). Microhardness measurements were carried out according to the diagram shown in Figure 3. A constant step from the surface after laser cutting or laser cutting and centrifugal shot peening was determined during the measurement. Measurements at a given depth were repeated 10 times. The maximum microhardness, which is just below the machined surface, was determined based on the micro hardness distribution. The results of the microhardness measurements served as a basis for calculating the degree of strain hardening of the surface layer e from the Equation (1):

(1) $$e=100\times \frac{{\mathrm{HV}}_{\max}-{\mathrm{HV}}_{0}}{{\mathrm{HV}}_{0}},\;\left(\%\right)$$

where:

${\mathrm{HV}}_{\max}$—maximum microhardness of the surface layer after centrifugal shot peening,
${\mathrm{HV}}_{0}$—microhardness before shot peening.

X-ray diffraction tests were performed with a Empyrean diffractometer (Panalytical, Malvern, UK). The experiments were carried out in the symmetrical Bragg-Brentano geometry, in which a divergent beam is used at room temperature and atmospheric pressure. Rectangular 5 × 8 × 15 mm^3^ specimens, which were cleaned in acetone prior to the experiments, were tested. A 3 kW copper anode lamp was used as the x-ray source. Anode radiation was filtered through nickel, which absorbs K_β_ radiation. Data were recorded in the angle range 2θ = 5–110° while using the step scan method. The scan step was 0.05° and the point exposure time was 3–5 s.

Residual stresses in the surface layer of laser-cut-and laser-cut and shot-peened specimens were tested while using the mechanical method. They were calculated on the basis of measurements of specimen deformations that occurred when the sequential layers of the material in which these stresses resided were removed.

## 3. Results

### 3.1. Surface Roughness 

**Figure 4:**
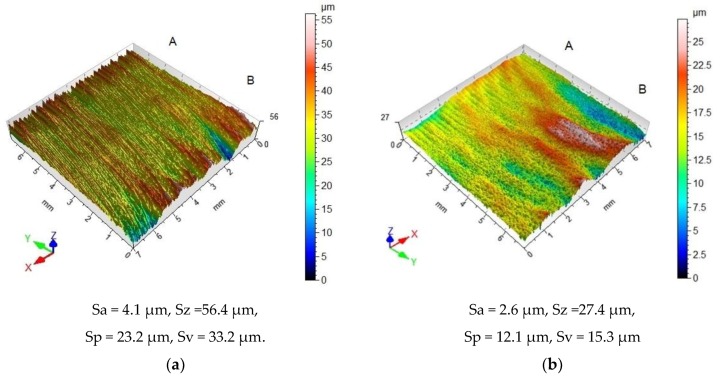
Surface topography C45 steel after: (**a**) laser cutting; (**b**) laser cutting and centrifugal shot peening (v_g_ = 1143 m/min, v_f_ = 3648 mm/min).

Figure 4a shows the surface topography of a laser-cut specimen. The surface formed by cutting is characterized by large differences in height (there are numerous peaks and valleys). In the area where the laser beam entered the material (A), the surface is covered with fine, evenly spaced straight drag lines. The surface roughness parameters for this area are as follows: Ra = 2.49 ± 0.12 µm and Rz = 15.76 ± 1.33 µm. In the laser beam exit zone (B), the drag lines are curvilinear; they deviate from standard slope (the intended path of the beam). This indicates that the material “slips away” from the kerf. The roughness parameters in the exit zone are Ra = 5.04 ± 0.35 µm and Rz = 31.29 ± 2.27 µm. The values of the surface roughness parameters in the exit zone are about twice as high as those in the entrance zone. The same relationship was observed for the parameters of the Abbott–Firestone curve, which were Rpk = 2.43 ± 0.3µm, Rk = 8.14 ± 0.38 µm, and Rvk = 3.72 ± 0.93 µm in the entrance zone and Rpk = 4.98 ± 0.58 µm, Rk = 16.54 ± 1.17 µm, and Rvk = 7.31 ± 0.56 µm in the exit zone. An increase in the viscosity of the workpiece at the bottom cut edge causes the large differences in the quality of the surface. This enhances the thickness of the liquid metal layer, which, as a result, sticks to the surface being cut. This promotes the formation of large irregularities. 

During centrifugal shot peening, the shot balls impact the surface of the laser-cut workpiece, leading to the deformation of its geometric structure (Figure 4b). As a consequence, the height of the drag lines is reduced and micro asperity summits are flattened. The difference between the height of the surface roughness profiles of the beam entrance zone (A) and the beam exit zone (B) is also reduced. 

**Figure 5:**
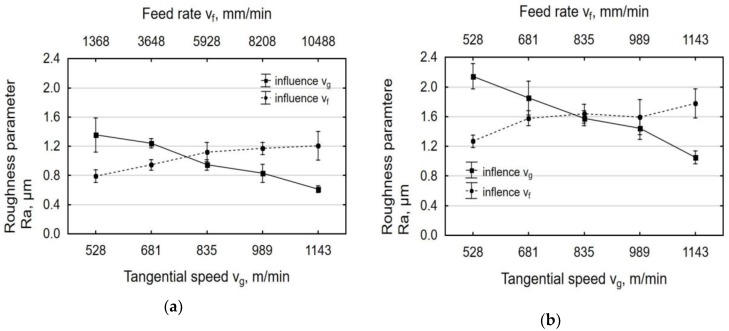
Effect of tangential speed v_g_ (v_f_ = 3648 mm/min) and feed rate v_f_ (v_g_ = 835 m/min) on the roughness parameter Ra specimens after laser cutting and centrifugal shot peening: (**a**) laser entrance zone; (**b**) laser exit zone.

Figure 5 shows the effect of tangential speed of the tool v_g_ and feed rate v_f_ on the roughness parameter Ra. As the rotational speed n, and thus tangential speed v_g_ increase, surface roughness decreases. This is due to the more intense smoothing of micro asperities on laser-cut surfaces as a result of increasing impact energy. The maximum decrease in Ra relative to the value recorded after laser cutting was four-fold. The changes in surface roughness that were obtained in the present study are more prominent when compared to those reported for vibratory-and-rotational shot peening of C45 steel specimens, for which a 30% reduction in surface roughness parameters [35] was observed, and for centrifugal shot peening of 41Cr4 steel specimens, where a three-fold reduction in Ra was obtained [26]. On the other hand, an increase in feed rate results in a decrease in the number of impacts per unit of exposure area, which leads to an increase in surface roughness. 

**Figure 6:**
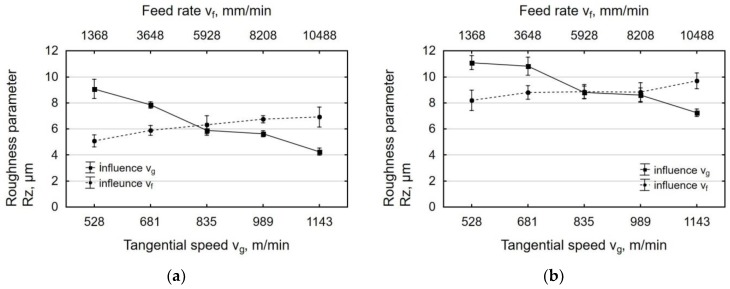
Effect of tangential speed v_g_ (v_f_ = 3648 mm/min) and feed rate v_f_ (v_g_ = 835 m/min) on the roughness parameter Rz specimens after laser cutting and centrifugal shot peening: (**a**) laser entrance zone; (**b**) laser exit zone.

The nature of the changes in the surface roughness parameter Rz as a function of the analyzed technological parameters of centrifugal shot peening is similar to that observed for Ra. An analysis of the changes in the parameter Rz of the roughness profile as a function of feed rate showed that the maximum reduction in Rz after centrifugal shot peening was about three-fold for the beam entrance zone and about four-fold for the exit zone (Figure 6).

**Figure 7:**
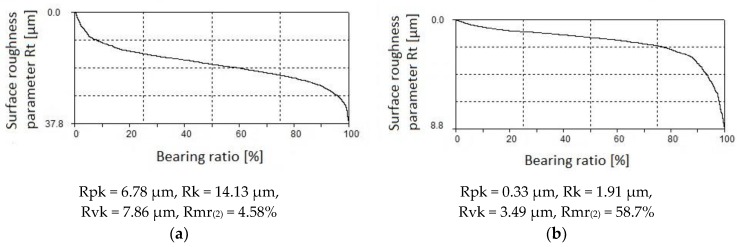
Curve of the material bearing for the “exit zone” after: (**a**) laser cutting; (**b**) laser cutting and centrifugal shot peening (v_g_ = 1143 m/min, v_f_ = 3648 mm/min).

Cooperation between two surfaces is described by the parameters that are associated with the Abbott–Firestone curve (bearing curve). The bearing curve for laser-cut parts is a degressive-progressive curve (Figure 7a). It has a large angle of inclination. The surface described by this curve has “sharp” micro-asperity summits and a small bearing area. The Abbott–Firestone curve for parts processed by centrifugal shot peening (Figure 7b) is only slightly inclined, which might point to a high abrasion resistance of the tested surfaces. It is a curve with a “thick center” and it can therefore be classified as a progressive curve.

**Figure 8:**
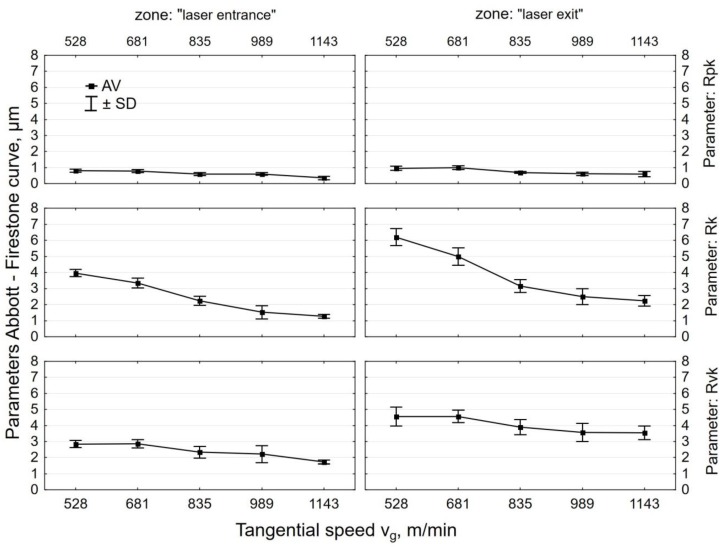
Effect of tangential speed v_g_ on the parameters Abbott-Firestone curve specimens after laser cutting and centrifugal shot peening (v_f_ = 3648 mm/min).

The effect of tangential speed v_g_ on the parameters of the bearing curve was similar to that observed for Ra and Rz (Figure 8). An increase in speed v_g_ caused more intense smoothing of micro asperities on the laser cut surface, which translated into a decrease in the values of Rpk, Rk, and Rvk parameters. Core roughness depth Rk, depending on the tangential speed of the peening head, decreased by two to seven times after shot peening. This means that centrifugal shot peening improves the so-called bearing capacity of the surface, or, to put it differently, that, after a grinding-in period, a substantial part of the surface will be in contact with the surface of the mating element. The Rpk parameter, which characterizes the behavior of a surface during grinding-in, decreased by 3 to 8.1 times relative to the value after laser cutting. The depth of the valleys (parameter Rvk) was reduced by centrifugal shot peening up to two times, depending on the tangential speed. This means that surfaces processed in this way may have lower oil retention capacity.

**Figure 9:**
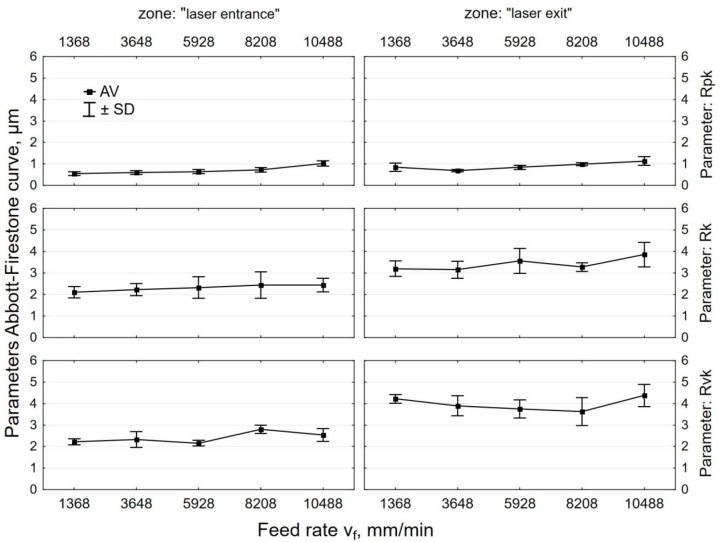
Effect of feed rate v_f_ on the parameters Abbott-Firestone curve specimens after laser cutting and centrifugal shot peening (v_g_ = 835 m/min).

The increase in the feed rate v_f_ (Figure 9) translated into a smaller proportion of elastic-plastic strains, which caused an increase in the parameters of the bearing curve. Feed rate v_f_ had less effect on the Abbott–Firestone curve parameters than tangential speed v_g_. At feed rate v_f_ = 1368 mm/min “(least)”, Rpk dropped up to six-fold when compared to the value that was obtained for laser-cut specimens. On the other hand, when a tangential speed v_g_ = 1143 m/min “(largest)” was used, an over eight-fold decrease in Rpk was observed. In the case of the roughness parameter Rk, the influence of tangential speed v_g_ was also more pronounced than the effect of feed rate v_f_. An up to five-fold reduction in Rk was obtained when compared to the value that was recorded for the laser cut specimens. After shot peening, parameter Rvk was reduced more than two-fold, a result that is similar to that obtained for tangential speed v_g_. 

**Figure 10:**
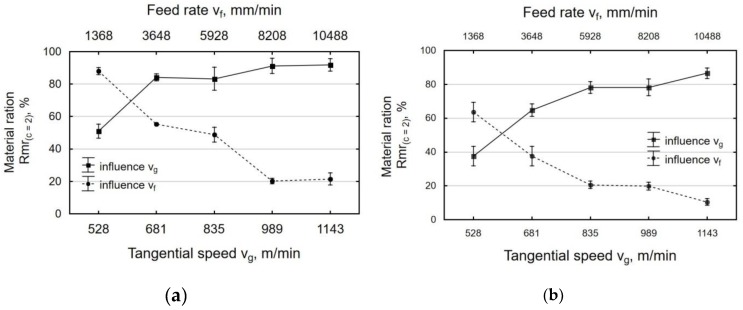
Effect of tangential speed v_g_ (v_f_ = 3648 mm/min) and feed rate v_f_ (v_g_ = 835 m/min) on the material ration specimens after laser cutting and centrifugal shot peening: (**a**) laser entrance zone; (**b**) laser exit zone.

The material ratio was increased for all of the investigated peening parameters when compared to the laser-cut surface (Figure 10). The material ratio of the roughness profile of the laser-cut parts was Rmr_(2)_ = 6.08% ± 0.57% for the beam entrance zone and Rmr_(2)_ = 5.42% ± 0.42% for the beam exit zone. This means that the laser-cut surface had low abrasion resistance. Centrifugal shot peening increased the material ratio by 3.33 to over 14 times for the beam entrance zone and up to almost 12 times for the beam exit zone. This suggests that the process also increased the abrasion resistance of the surface.

During centrifugal shot peening, there is a wide angle between the normal velocity vector and the peened surface. This promotes strong friction between the peened surface and the shot, which causes intense abrasion of the micro-asperity summits that previously formed on the hard material, decreasing the height of micro asperities and leading to the formation of micro-cavities on the peened surface. The favorable changes in the stereometric properties of the peened surface are likely to make the surface resistant to abrasive wear [35]. The “smoothing” of the surface as a result of deformation leads to an evening out of zones with varying surface roughness, which is likely to have a positive effect on the use of a given part in a next production stage (e.g. application of paint coatings). 

### 3.2. Microstructure and Microhardness

**Figure 11:**
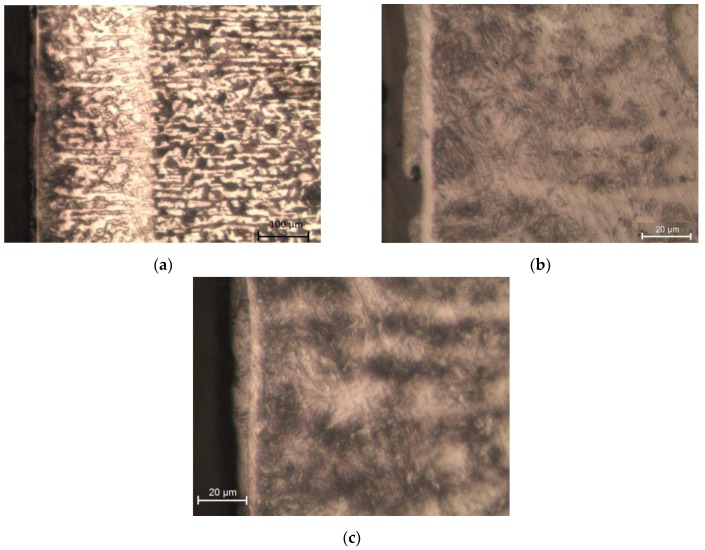
Microstructure of C45 steel: (**a**) HAZ and transition zone; (**b**) HAZ after laser cutting; (**c**) HAZ after laser cutting and centrifugal shot peening (v_g_ = 1143 m/min, v_f_ = 3648 mm/min).

Before the laser cutting process, the C45 steel specimens had a ferritic–pearlitic structure. As a result of strong heating with the laser beam followed by cooling down, the material around the cut edge became hardened. A heat affected zone was created (Figure 11a), which differed in the structure and properties from the parent material. In the heat affected zone, martensite needles (Figure 11b) are visible next to the edge of the cut, with ferrite, and then martensite located further away from the edge. The laser cutting process lasted too short for ferrite grains to become enriched with carbon, which resulted in the formation of low-carbon martensite. Perlite, on the other hand, transformed into high carbon martensite. The cyclic bombarding of the material with shot particles modifies its structure (Figure 11c). The deformation of the material caused by centrifugal shot peening leads to an increase in dislocation density. The gliding dislocations are stopped when they encounter obstacles, such as other dislocations, grain boundaries, or cementite separation. The increase in the dislocation density has a hardening effect on the material. The resulting structure can be classified as fine-needle martensite.

**Figure 12:**
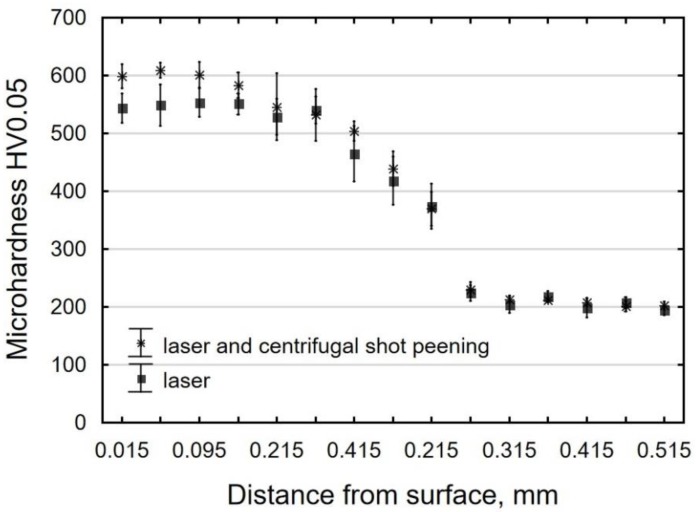
Microhardness distribution of C45 steel element surface layer after laser cutting and laser cutting and centrifugal shot peening (v_g_ = 1143 m/min, v_f_ = 3648 mm/min).

In laser-cut specimens, the zone of changes in microhardness is over 0.3 mm wide (Figure 12). The microhardness of the surface layer increased more than twice in the area of the cut edge (approx. 560 HV0.05), and then slowly decreased to reach the core microhardness value (approx. 200 HV0.05). The literature only offers reports on the use of centrifugal shot peening for processing parts with hardnesses of 240 HV and 360 HV [26]. However, the present study shows that the laser-cut parts can also be treated by centrifugal shot peening. Though the hardness and thickness of the hardened layer only increase slightly during centrifugal shot peening (Figure 12), the increase is likely to translate into increased resistance to abrasive wear [35].

**Figure 13:**
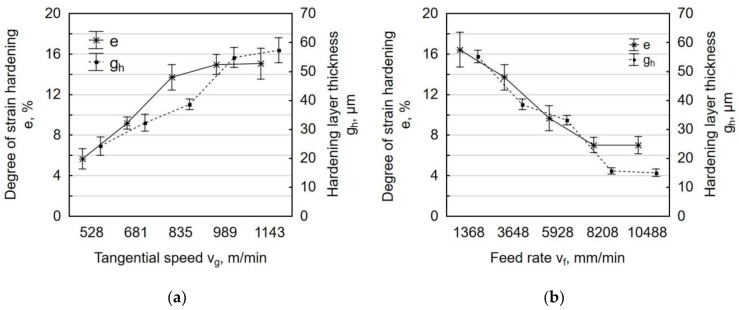
Degree of strain hardening e and hardening layer thickness g_h_ specimens after laser cutting and centrifugal shot peening as a function of: (**a**) tangential speed v_g_ (v_f_ = 3648 mm/min); (**b**) feed rate v_f_ (v_g_ = 835 m/min).

The degree of strain hardening of the surface layer for laser-cut-and shot-peened C45 steel depend on the technological parameters of shot peening (Figure 13). An increase in the tangential speed v_g_ leads to an increase in impact energy and it elevates the number of impacts per unit of area, which translates into a greater proportion of plastic-elastic deformations in the workpiece and thus increases the degree of strain hardening of the surface layer. The thickness of the hardened layer g_h_, in relation to the core, was max. 60 µm, as verified by a statistical test of means. The largest changes in microhardness occurred right next to the treated surface, where the most deformed grains were found (Figure 11c). The degree of strain hardening increased as a function of tangential speed v_g_ from about 6% to 15% (Figure 13a), with the dynamics of increase the degree of strain hardening declining in the velocity range v_g_ = 989–1143 m/min.

A rise in feed rate increases the spacing between the indentations that are imparted by shot, which results in uneven deformation of the treated surface. The changes in the thickness of the hardened layer after centrifugal shot peening (for a variable feed rate v_f_) ranged from 15 µm to 56 µm. At low feed rates, the number of impacts per unit area was higher, which increased the thickness of the hardened layer. The increase in microhardness for v_f_ = 1368 mm/min relative to the value that was recorded after laser cutting was 16.4% (Figure 13b).

### 3.3. XRD Tests

**Figure 14:**
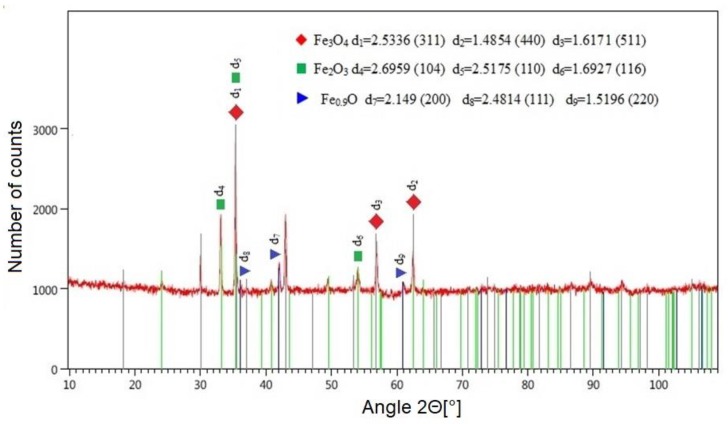
Diffraction pattern with matching theoretical reflection obtained after analyzing the surface of C45 steel after laser cutting.

The presence of oxygen as the assist gas during cutting promotes an exothermic reaction, which leads to the formation of combustion products on the cut surface. This is confirmed by the qualitative change in the phase composition of the surface layer after laser cutting (Figure 14). The XRD tests that we carried out allowed for us to identify the following phases on the surface of C45 steel: Fe_3_O_4_, Fe_2_O_3_, Fe_0.9_O (Figure 14). Table 2 shows the lattice parameters and angles for the compounds formed on the surface of metal as a result of laser cutting. 

**Figure 15:**
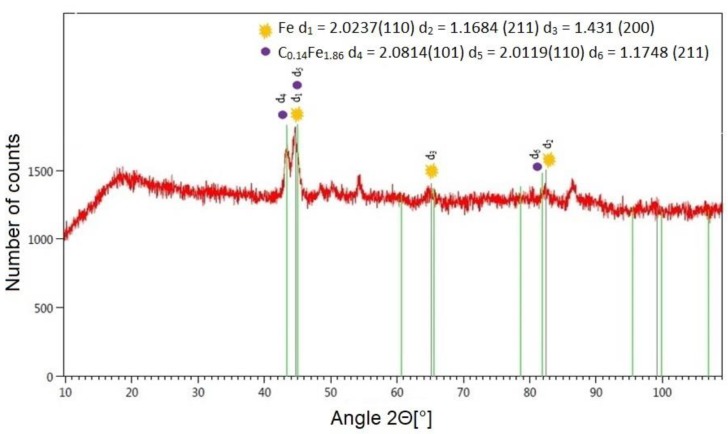
Diffraction pattern with the matching theoretical reflection obtained after analyzing surface C45 steel after laser cutting and centrifugal shot peening (v_g_ = 835 m/min, v_f_ = 3648 mm/min).

XRD tests of a laser-cut-and shot-peened specimen demonstrated the presence of two compounds on the treated surface. The diffractogram (Figure 15) shows two main dominant peaks, which correspond to ferrite and martensite in the diffraction database (Table 3). Shot balls, when impacting the surface of the workpiece, “blast off” and “shear off” oxide phases, removing this way the thin oxide film that had formed on the surface of the workpiece. The removal of combustion products from the treated surface reduces or eliminates the peeling of coatings applied to it.

### 3.4. Residual Stress

**Figure 16:**
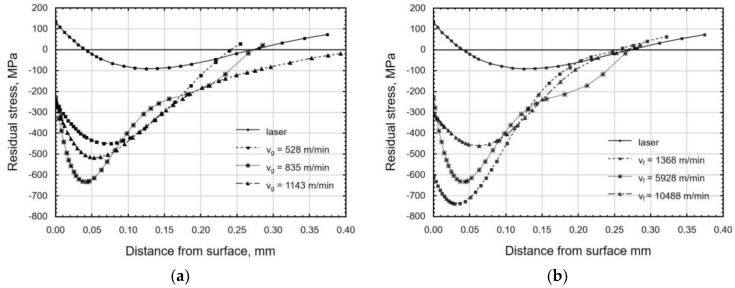
Distribution of residual stress in the function distance from sample surface after laser cutting and centrifugal shot peening at the varible: (**a**) tangential speed v_g_ (v_f_ = 5928 mm/min); (**b**) feed rate v_f_ (v_g_ = 835 m/min).

Laser cutting is accompanied by the re-solidification of molten material, which takes place in unstable conditions. This promotes the occurrence of tensile residual stresses that reside in the specimens at a depth of about 0.05 mm from the surface of the workpiece (Figure 16). Tensile stresses also originate from the structural changes that take place during the cutting process.

In the process of centrifugal shot peening, compressive residual stresses are produced in the surface layer (Figure 16), which improve the functional properties of the material, e.g. increase its resistance to fatigue wear [34]. Grinding, which has been used so far to finish laser cut parts, leads to the formation of tensile residual stresses in the surface layer [22].

An increase in the tangential speed of the tool increased the depth of compressive residual stresses (Figure 16a), and also, though to a lesser extent, contributed to an increase in the value of these stresses. The absolute maximum value of compressive residual stress was found at a depth of 0.05–0.1 mm. On the other hand, as the feed rate increased, the absolute maximum value of compressive stresses produced in the peened part decreased. The depth of compressive residual stresses did not change significantly (Figure 16b). The lowest residual stress was obtained at v_f_ = 10 488 mm/min and v_g_ = 835 m/min within the range of the technological parameters of the experiment.

## 4. Conclusions

The following conclusions can be drawn based on the centrifugal shot peening tests of the laser-cut C45 steel elements.

1. The use of centrifugal shot peening for finish machining of laser-cut C45 steel parts allowed for obtaining a four-fold reduction in the surface roughness parameters Ra and Rz. Centrifugal shot peening diminished the differences in Ra and Rz between the beam entrance zone and the beam exit zone.
2. An increase in the tangential speed of the tool v_g_ resulted in a decrease in roughness parameters, while an increase in feed rate v_f_ had the opposite effect, with the changes being more pronounced for the variable v_g_.
3. Centrifugal shot peening allowed for changing the nature of the bearing curve from a degressive–progressive curve to a degressive curve. A significant decline in Rpk, Rk, and Rvk parameters was observed as compared to the values that were obtained after laser cutting.
4. Centrifugal shot peening resulted in an up to 14-fold increase in the material ratio of the roughness profile as compared to the value obtained after laser cutting.
5. Peened workpieces had an up to 16% higher microhardness (v_g_ = 835 m/min, v_f_ = 1365 mm/min) and a 15 μm to 58 μm deep hardened layer.
6. As the surface of a workpiece was impacted by shot during centrifugal shot peening, oxide phases, which are combustion products, were blasted off and sheared off the surface, which caused the removal of Fe_3_O_4_, Fe_2_O_3_, and Fe_0.9_ oxides.
7. In the surface layer of the specimens, compressive residual stresses were formed during centrifugal shot peening, whose absolute maximum value varied from 450 MPa to 740 MPa. The stresses resided at a depth of 0.25–0.40 mm, depending on the technological parameters of the peening process.
8. As the tangential speed of the peening head v_g_ grew to 835 m/min, an increase was observed in the absolute value of compressive residual stresses and the depth of their accumulation; a further increase in tangential speed only increased the depth of the occurrence of compressive residual stresses. An increase in feed rate v_f_ caused a decrease in the absolute value of compressive residual stresses, but it did not significantly affect the depth of accumulation of residual stresses.
9. The results of the centrifugal shot peening experiments demonstrate that this cold working method can be successfully used with CNC machine tools. The CNC machine tools are equipped with control systems that can be used to guide the tool along a designated path, which allows for processing parts with complex shapes.

## Figures and Tables

**Table 1:** Chemical composition and selected properties of C45 steel.

Chemical Composition, (%)								
C	Mn	Si	P	S	Cr	Ni	Mo	Fe
0.48	0.74	0.36	0.011	0.01	0.09	0.02	0.002	rest
Yield point						R_e_ = 430 MPa		
Tensile strength						R_m_ = 740 MPa		
Hardness						250 HB		

**Table 2:** Data of identified compounds based on the diffraction pattern obtained after analysis surface of C45 after laser cutting.

Number in the Base ICDD PDF–4+	Chemical Formula	Lattice Parameters (nm)	Angles (°)
04-009-8436	Fe_3_O_4_	a = b = c = 0.8403	α = β = γ = 90°
04-006-9058	Fe_2_O_3_	a = b = 0.5350 c = 1.3720	α = β = 90° γ = 120°
04-001-9267	Fe_0.9_O	a = b = c = 0.4298	α = β = γ = 90°

**Table 3:** Data identified phases based on the diffraction patter obtained after analysis surface of C45 after laser cutting and centrifugal shot peening.

Number in the Base ICDD PDF–4+	Chemical Formula	Lattice Parameters (nm)	Angles (°)
04-002-1061	Fe–α	a = b = c = 0.2862	α = β = γ = 90°
00-044-1289	C_0.14_Fe_1.86_	a = b = 0.2846 c = 0.3053	α = β = γ = 90°
